# Supplementary material for: Phytochemicals from Vanda bensonii and Their Bioactivities to Inhibit Growth and Metastasis of Non-Small Cell Lung Cancer Cells
Source: Molecules. 2022 Nov 15;27(22):7902. doi: 10.3390/molecules27227902 (PMC9699248; doi:10.3390/molecules27227902)

Supplementary Materials

Phytochemicals from *Vanda bensonii* and Their Bioactivities to Inhibit Growth and Metastasis of Non-Small Cell Lung Cancer Cells

Tajudeen O. Jimoh [1,2], Narawat Nuamnaichati [3], Rungroch Sungthong [3], Chaisak Chansriniyom [4,5], Pithi Chanvorachote [6,7], Kittisak Likhitwitayawuid [4], Chatchai Chaotham [3,7,*] and Boonchoo Sritularak [4,8,*]

1 Pharmaceutical Sciences and Technology Program, Faculty of Pharmaceutical Sciences, Chulalongkorn University, Bangkok 10330, Thailand

2 Department of Biochemistry, Faculty of Health Sciences, Islamic University in Uganda, Kampala P.O. Box 7689, Uganda

3 Department of Biochemistry and Microbiology, Faculty of Pharmaceutical Sciences, Chulalongkorn University, Bangkok 10330, Thailand

4 Department of Pharmacognosy and Pharmaceutical Botany, Faculty of Pharmaceutical Sciences, Chulalongkorn University, Bangkok 10330, Thailand

5 Natural Products and Nanoparticles Research Unit, Chulalongkorn University, Bangkok 10330, Thailand

6 Department of Pharmacology and Physiology, Faculty of Pharmaceutical Sciences, Chulalongkorn University, Bangkok 10330, Thailand

7 Center of Excellence in Cancer Cell and Molecular Biology, Pharmaceutical Sciences, Chulalongkorn University, Bangkok 10330, Thailand

8 Center of Excellence in Natural Products for Ageing and Chronic Diseases, Faculty of Pharmaceutical Sciences, Chulalongkorn University, Bangkok 10330, Thailand;

* Correspondence: cchoatham@gmail.com (C.Chaotham); Tel.: (+66 (0) 2218 8372), boonchoo.sr@chula.ac.th (B.S.); Tel. (+66 (0) 2218 8356)

Figure S1. Sigmoidal dose-response plots of NCI-H460 cells treated with varying concentrations of *Vanda bensonii* metabolites. Percentages of cell viability after treating cells with varying concentrations of *V. bensonii* metabolites for 24 h, assessed by a colorimetric method. All experiments were done at least in triplicates, and 0.5% (v/v) dimethyl sulfoxide served as the vehicle control. The graphical results represent means ± standard deviations.

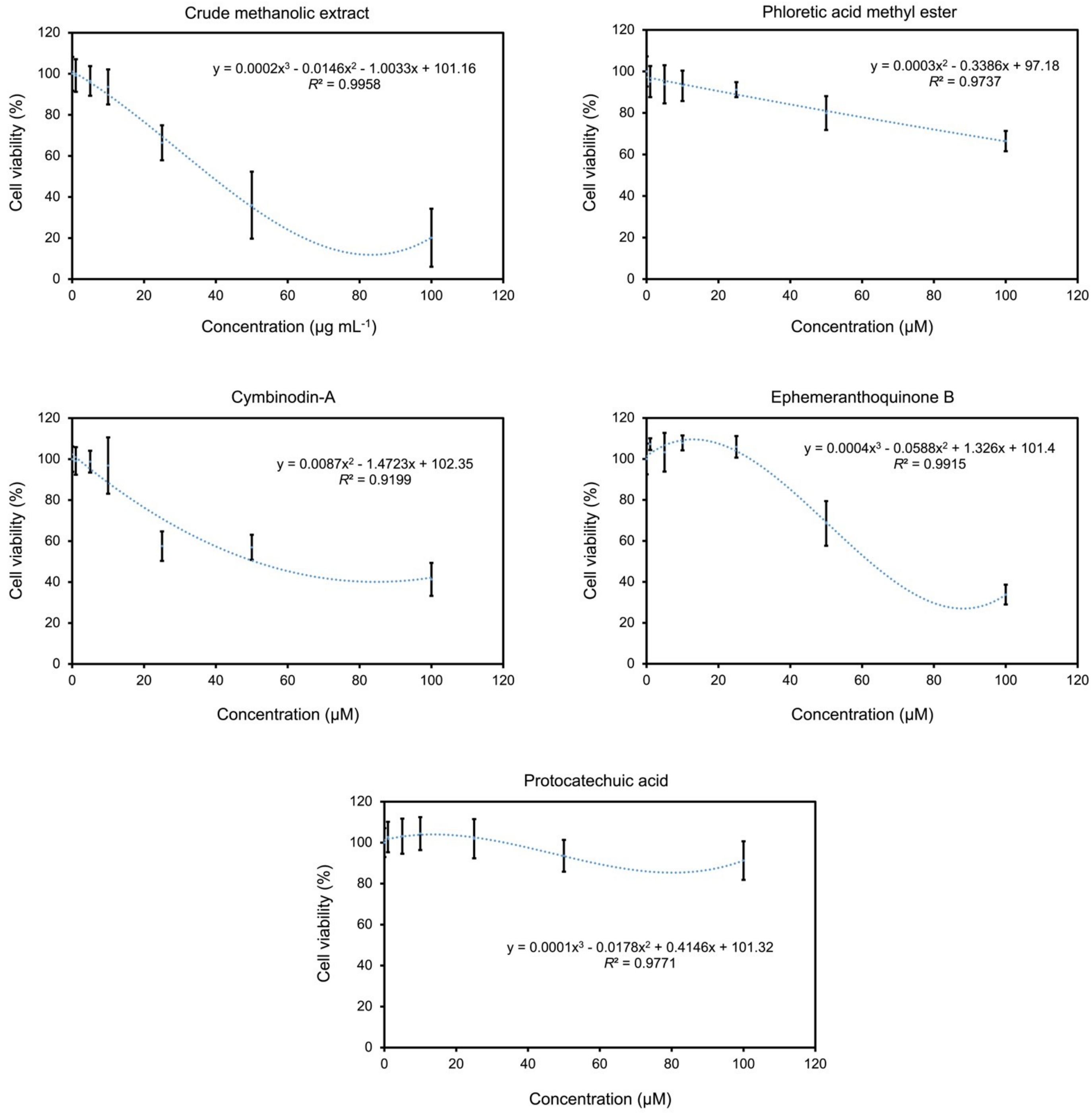

Supplement: Supplementary file 1 [file molecules-27-07902-s001.zip › molecules-2011422-supplementary.pdf]
